# Reconstructing for joint angles on the shoulder and elbow from non-invasive electroencephalographic signals through electromyography

**DOI:** 10.3389/fnins.2013.00190

**Published:** 2013-10-24

**Authors:** Kyuwan Choi

**Affiliations:** Psychology Department, Computational Biomedicine Imaging and Modeling, Computer Science, Rutgers UniversityPiscataway, NJ, USA

**Keywords:** EEG, EMG, neural activity, primary motor cortex

## Abstract

In this study, first the cortical activities over 2240 vertexes on the brain were estimated from 64 channels electroencephalography (EEG) signals using the Hierarchical Bayesian estimation while 5 subjects did continuous arm reaching movements. From the estimated cortical activities, a sparse linear regression method selected only useful features in reconstructing the electromyography (EMG) signals and estimated the EMG signals of 9 arm muscles. Then, a modular artificial neural network was used to estimate four joint angles from the estimated EMG signals of 9 muscles: one for movement control and the other for posture control. The estimated joint angles using this method have the correlation coefficient (CC) of 0.807 (±0.10) and the normalized root-mean-square error (nRMSE) of 0.176 (±0.29) with the actual joint angles.

## Introduction

The field of Brain Machine Interface (BMI) has engaged in active research to help paralyzed patients regain some independence and to better integrate within societal activities. Brain-machine interface can be broadly divided into invasive and non-invasive modalities depending on how brain signals are harnessed. Invasive BMI, mainly targets motor related cortical areas. Non-human primates are often used to for example, harness the spikes and local field potentials from the primary motor cortex, known to interface with the spinal cord and containing signals useful to control arm movement. Such neural signal has been used to control external devices such as a robotic arm or a mouse cursor by reconstructing hand trajectories from the measured neural activities (Chapin et al., 1999; Wessberg et al., 2000; Serruya et al., 2002; Talylor et al., 2002; Carmena et al., 2003). More recently invasive BMI has been approved to be used in humans (Hochberg et al., 2006; Chadwick et al., 2011), including as well electrocorticography (ECoG) (Schalk et al., 2007; Sanchez et al., 2008).

In the case of non-invasive BMI, the state-of-the-art research uses motor imagery, a paradigm that classifies whether the subject performs left or right hand motor imagery using electroencephalography (EEG) signals (Ramoser et al., 2000; Wolpaw and McFarland, 2004). It was believed that the noisy EEG signal in non-invasive BMI would be insufficient to estimate three-dimensional hand movement (Lebedev and Nicolelis, 2006). However, recently Bradberry et al. (2010) succeeded in reconstructing the three-dimensional hand movements from the EEG signals while the subjects perform natural and self-initiated reaching actions.

In the present study, a new method using electromyography (EMG) signals is proposed that first reconstructs the EMG signals of the arm muscles from the source currents estimated from EEG signals, and then estimates joint angles on the shoulder and the elbow from the reconstructed EEG signals. By reconstructing the EMG signals of the arm muscles from EEG signals, it is possible to reconstruct not only kinematics-, but also dynamics-based information involving force generation. For example, impedance and joint torque can be obtained to build more realistic brain-machine interfaces, compatible with human motion execution. Furthermore, when reconstructing EMG signals from the EEG signals, by electrically stimulating the arm muscles of a paralyzed person, it is possible using a functional electrical stimulation (FES) system to engage the person in self-adaptive control of his/her arm.

In this study, source currents over 2240 vertexes were estimated from EEG signals of 64 channels through a hierarchical Bayesian method introducing a hierarchical prior (Sato et al., 2004). This method can effectively incorporate both structural and functional MRI data. In this hierarchical Bayesian method, the variance of the source current at each source location is considered an unknown parameter and estimated from the observed EEG data and prior information by using the Variational Bayesian (VB) method. The fMRI information was imposed as prior information on the variance distribution rather than the variance itself so that it gives a soft constraint on the variance. From the estimated source currents over 2240 vertexes, only 33 vertexes are selected, which is located in the left primary motor cortex contralateral to moving arm, to estimate the filtered EMG signals of 9 muscles by using a sparse linear regression method which can automatically select only useful features in estimating the filtered EMG signals. A modular artificial neural network was then used to reconstruct 4 joint angles on the shoulder and elbow from the estimated filtered EMG signals, which trains movement data and posture data in two different networks. This modular structure improves the accuracy of the estimation.

## Materials and methods

### Experimental task

**Figure 1:**
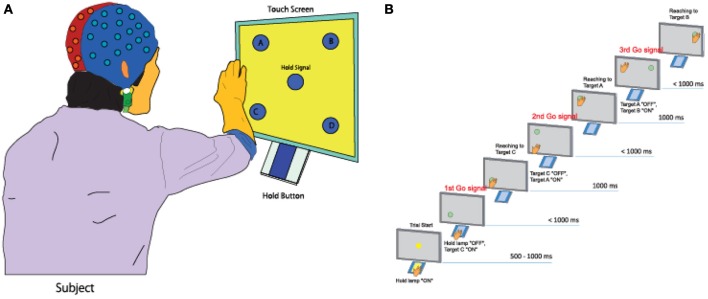
**Experimental task. (A)** A subject performs a continuous arm-reaching task while facing a touch screen displaying five lights and five buttons. **(B)** Sequential arm-reaching task (Hold-C-A-B sequence).

Five healthy right-handed subjects (five men, Mage = 22.51, age range: 20–29 years) participated in the experiment. All five subjects do not have any experience of participating in the experiments of brain-machine interface study before. All participated subjects submitted a written form of consent before starting the experiment. The subjects performed a continuous arm-reaching task as shown in Figure 1A. The task consisted of pushing buttons in the following sequences: Hold-C-A-B, Hold-C-D-B, Hold-D-B-A, and Hold-D-C-A. Theses sequences are explained in greater detail below.

Here only the Hold-C-A-B sequence is explained (Figure 1B), since the others have similar patterns. First, a subject pushes the hold button for 1 s when the hold signals turns on. If the subject succeeds in pushing the hold button for 1 s, the C button turns on, and the hand of the subject has to move to the C button within 1 s. If the subject is successful in keeping the C button pressed for 1 s, the A button turns on. The hand of the subject is then supposed to push the A button within 1 s and keep it pressed for 1 ṡ. If the A button is successfully pressed for 1 s, the B button turns on. The hand of the subject then has to push the B button within 1 s and keep it pressed for 1 s.

When the subject succeeded in pressing all three buttons correctly, it was considered as success, and only successful trials were analyzed in this study. After pressing the three buttons, the subject takes a rest between 3 and 4 s, then, it goes to the next trial. The task of the next trial is decided randomly. By performing 10 trials for one of four tasks randomly, each subject conducted 40 trials within one set. A total of seven sets of experiment were conducted for one subject. The leave-one-out cross-validation method was used to analyze the measured data by using six sets for training data and one set for the test data.

### fMRI experiment

**Figure 2:**
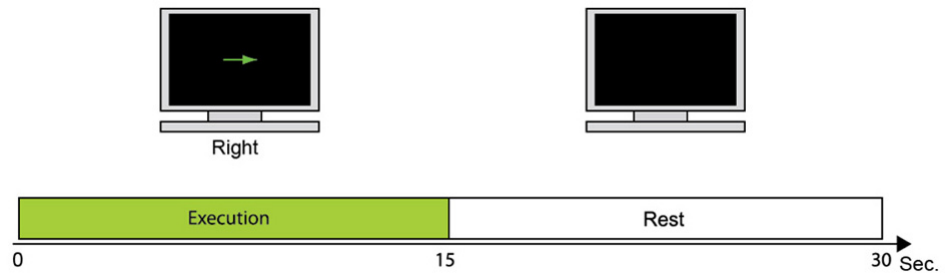
**fMRI task**. During 15 s, the participant moves the right index finger in the instructed direction every 1 s. The monitor then goes blank and the participant takes a break for 15 s while watching the monitor.

Figure 2 shows the fMRI task used to collect fMRI data as prior information to estimate cortical activity. One trial consisted of the execution task in which the participant moves the right index finger (e.g., up or down) every 1 s. This is followed by a resting period in which the participant takes a break for 15 s. Each participant conducted 24 trials of the fMRI task. The fMRI activity when participants take a rest (rest periods) was subtracted from the fMRI activity when participants moved their fingers (execution periods). All five participants conducted the fMRI task to get their individual prior information.

### Estimation of cortical activities from EEG signals

EEG signals were measured at 1 kHz sampling rate on 64 channels by using a biosemi system (Amsterdam, Netherlands). The measured EEG signals were taken baseline corrected (baseline data from −1 to 0 s) and band-pass filtered between 8 and 30 Hz using a fifth-order butterworth filter.

To estimate cortical activities from EEG signals, an inverse filter L (a matrix of dimensions 2240 × 64) in Equation 1 was used. By multiplying real-time EEG signals to the obtained inverse filter as in Equation 1, it is possible to quickly estimate the cortical activity.

(1) $$\begin{array}{l} L\left(\sum_{\alpha }^{-1}\right)=\sum_{\alpha }^{-1}\cdot \;G^{\prime }\;\cdot \;{\left(G\cdot \sum_{\alpha }^{-1}\cdot \;G^{\prime }+\beta^{-1}I_{M}\right)}^{-1},\\ \;J(t)\;=L\left(\sum_{\alpha }^{-1}\right)\;\cdot \;E(t) \end{array}$$

Here, *E*(*t*) represents measured real-time EEG signals given by 64 × 256 Hz (sampling rate). *J*(*t*) denotes the estimated cortical activities over 2240 vertexes every second and is given by 2240 × 256 Hz entries. *G* (64 × 2240) is a lead field matrix which represents the impulse response of each source vector component at every measurement site (Baillet et al., 2001) and G′ denotes its transpose. The boundaries between brain, skull, and scalp were generated by using the Curry 5 software (Compumedics, USA). Here, the relative conductivities of the brain, skull, and scalp are 10.0125 and 1. *I*_*M*_ represents an identity matrix of M-by-M (M:number of sensors), β^−1^ (64 × 64) corresponds to the inverse of the noise variance of the observed EEG signals. ∑^−1^_α_ denotes the source covariance matrix, and is calculated as ∑^−1^_α_ =diag(α^−1^). Here, α^−1^ (2240 × 2240) represents the source current variance which is considered unknown parameters in this study and estimated from the measured EEG data by applying a hierarchical prior on current variance.

Artifact dipoles were also incorporated in the estimation according to previous studies (Fujiwara et al., 2009; Morishige et al., 2009). Artifact dipoles were located at the center of the heart, the right shoulder, and wrist joints, the left and right eyeballs, and the carotid arteries, and estimated.

#### Estimation of current variance

In this study, the current variance α^−1^ was estimated by the Automatic relevance determination (ARD) hierarchical prior (Neal, 1996).

(2) $$\begin{array}{l} P\text{​}\left(J(t)|\alpha ,\beta \right)\propto \exp\text{​}\left[-\frac{\beta }{2}J^{\prime }(t)\cdot A\cdot J(t)\right]\\ \;P\text{​}\left(\alpha_{i}\right)=\Gamma \text{​}\left(\alpha_{i}|\alpha_{0i},r_{0}\right),\\ \;P\text{​}\left(\beta \right)=\frac{1}{\beta } \end{array}$$

where β is the inverse noise variance of the observed EEG signals, *A* = diag(α), and α is an I-by-1 vector whose component α_*i*_ is the inverse current variance corresponding to the *i*-th current dipole. Γ represents the Gamma distribution with mean α_0*i*_ and degree of freedom *r*_0_. Intuitively, the hyper-parameter *r*_0_ represents confidence of the hierarchical prior information. A prior current variance *v*_0*i*_ =α^−1^_0*i*_ represents the prior information on current intensity. For large and small *v*_0*i*_, estimated current *J*_*i*_ (*t*) tends to be large and small, respectively. These values were determined from the fMRI information:

(3) $$v_{0i}=v_{\text{base}}+\left(m_{0}-1\right)\cdot v_{\text{base}}\cdot {\left({\hat{t}}_{i}\right)}^{2},$$

where ${\hat{t}}_{i}$ is a normalized *T*-value on the *i*-th vertex. Normalized *T*-values are computed by dividing the original *T*-values by the maximum of those *T*-values (thus ranging from 0 to 1).

*v*_base_ is a baseline of the current variance, which is estimated from the pre-movement interval (1.0–0.5 s before the movement initiation) of the EEG data by a Bayesian minimum norm estimation. A variance magnification parameter *m*_0_, which is the other hyper-parameter, specifies the scaling between the current variances in the baseline and task periods. *m*_0_ = 100 and *r*_0_ = 10 were used.

Due to the hierarchical prior, the estimation problem becomes non-linear and cannot be solved analytically. Therefore, the VB method (Attias, 1999; Sato, 2001) is employed. In the VB method, *J*(*t*), α, and β are iterately updated until convergence.

**Figure 3:**
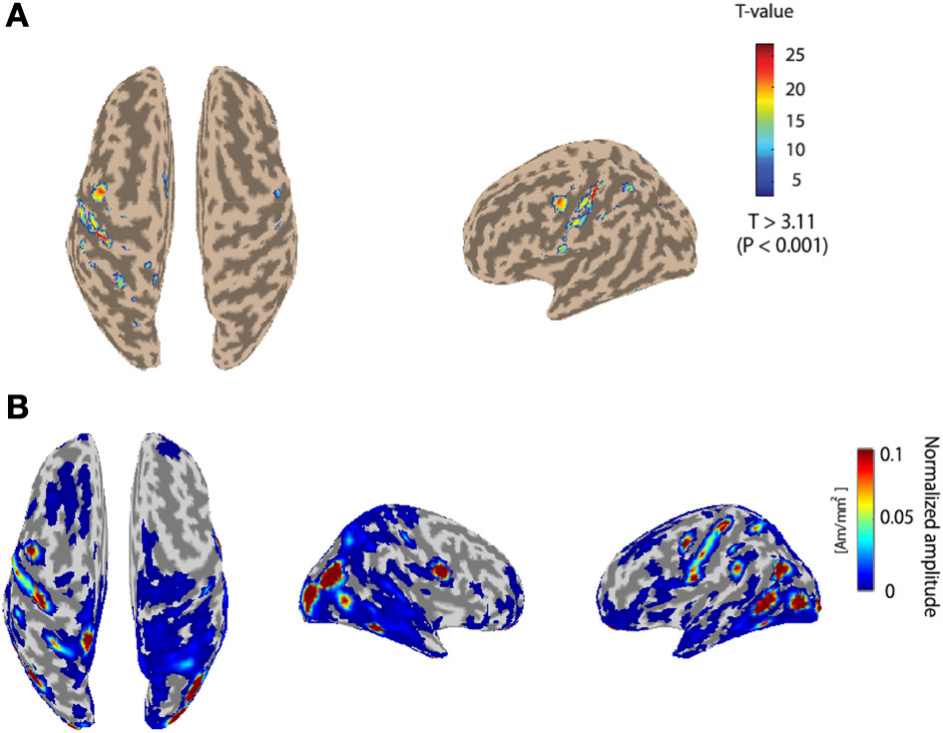
**(A)** The fMRI activity while subject 1 performs the Hold-C-A-B sequence task. **(B)** Estimated cortical activities of subject 1 for the task of the Hold-C-A-B sequence (an average of 70 trials).

Figure 3A depicts the fMRI activity while subject 1 conducts the Hold-C-A-B sequence task with the right arm. The left primary motor area is strongly activated. The fMRI information was used as the prior information to estimate cortical activities. Figure 3B shows the cortical activities of subject 1 estimated from the EEG signals for the Hold-C-A-B task. As expected, strong cortical activities are estimated in the left motor cortex. Meanwhile, several parts in the visual cortex are activated in the figure. The reason of the activation of the visual cortex is that while the subject performs the task of the experiment, he sees the target buttons emitting high intensity light.

### EMG signal processing

**Figure 4:**
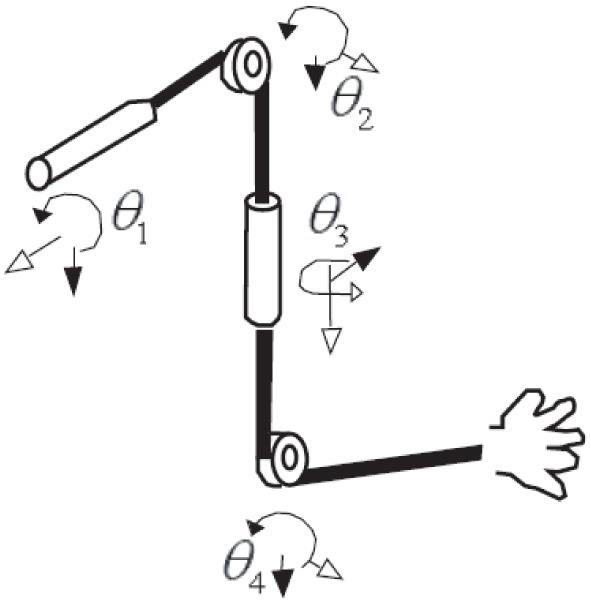
**Four-degrees-of-freedom arm movement**.

For all trials in this study, EEG, EMG signals, and the positions of the shoulder, the elbow, and the wrist of the subject were simultaneously measured. EMG signals were collected in the nine muscles involving four degrees of freedom (see Figure 4 and Table 1).

**Table 1:** **Muscles measured for EMG signals**.

θ_1_	Adduction	Pectoralis major, Teres major
	Abduction	Deltoid, Deltoideus
θ_2_	Extension	Deltoid, Teres major, Triceps Brachii C. L., T. B. C. Laterale
	Flexion	Deltoid, Pectoralis major, Biceps Brachii, Deltoideus
θ_3_	Medial rotation	Deltoid, Pectoralis major, Teres major, Deltoideus
	Lateral rotation	Deltoid, Infraspinatus, Deltoideus
θ_4_	Extension	Triceps Brachii Caput Longus, Triceps Brachii Caput Laterale
	Flexion	Biceps Brachii, Brachialis

In order to measure the EMG signals, a silver/silver chloride surface electrode (NE-102, Nihon Kohden) was used. After differential amplification, each signal was sampled at 1 kHz with a 12-bit resolution. The signals were digitally rectified, averaged over 5 ms, and then filtered through a second-order low-pass filter with a cut-off frequency of ~3 Hz (Koike and Kawato, 1995).

(4) $$f_{\text{EMG}}(t)=\sum_{j=\;1}^{n}h_{j}\text{EMG}(t-j+1),$$

(5) $$h(t)=6.44\times \left(\exp^{-10.80t}-\exp^{-16.52t}\right)\text{​},$$

The coefficient *h*_*j*_ in Equation 4 can be acquired by sampling *h*(*t*) in Equation 5 discretely. The resulting signal is very similar to the actual tension; consequently, it is called quasi-tension (Basmajian and DeLuca, 1985).

The method that uses a low-pass filter to estimate muscle tension shows good performance when the velocity of muscle contraction is slow. However, the method cannot estimate muscle tension precisely when the velocity of contraction is very high, and the method does not consider the non-linear characteristics of muscles, such as length and velocity. However, it is reasonable to assume that the output of the low-pass filter is similar to the actual tension (Mannard and Stein, 1973).

### Kinematics

In order to measure the position of the shoulder, the elbow, and the wrist of the subjects, an infrared marker was attached on their arms and measured each position by using a 3D position measurement system (MacReflex, Qualisys). The sampling rate was 120 Hz. In order to calculate the joint angles of the four degrees of freedom in the shoulder and elbow from the positions measured, the inverse kinematics equations (Koike and Kawato, 1994) was used.

**Figure 5:**
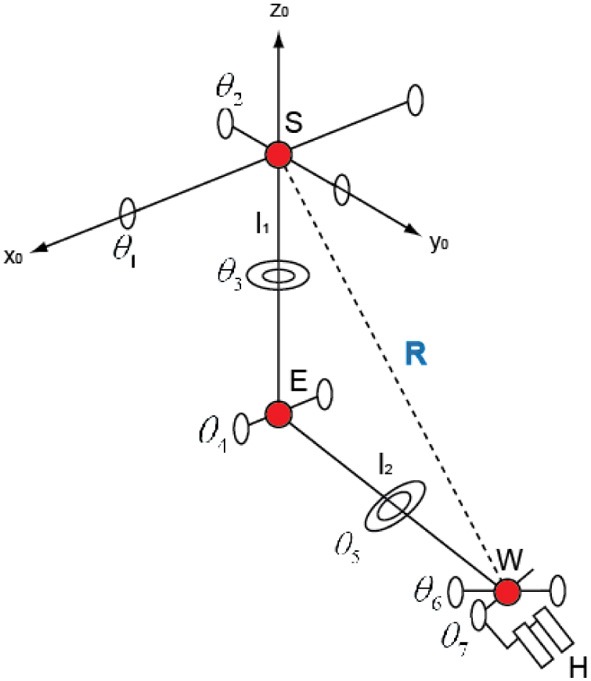
**The structure of the joints of a human**.

In Figure 5, if we set the transition matrix of θ_1_ θ_2_, …,θ_7_ to *A*_*x*_ (θ_1_), *A*_*y*_ (θ_2_), …, *A*_*z*_(θ_7_) and the transition matrix of *l*_1_ (the length of the upper arm), *l*_2_ (the length of the fore arm), and *l*_3_ (the length of the hand) to *L*_*z*_ (*l*_1_), *L*_*z*_ (*l*_2_), and *L*_*z*_ (*l*_3_), we can represent the transition matrix of *A*_*E*_, *A*_*W*_, and *A*_*H*_, which represents the relation from the elbow position *E* to the hand position *H*, like below,

(6) $$A_{E}=A_{x}\left(\theta_{1}\right)A_{y}\left(\theta_{2}\right)L_{z}(l_{1})=\left[\begin{array}{cc} C_{2} & C_{2}l_{zl}\\ 0^{T} & 1 \end{array}\right],$$

Here,

(7) $$C_{2}=C_{x}\left(\theta_{1}\right)C_{y}\left(\theta_{2}\right)=\left[\begin{array}{ccc} 1 & 0 & 0\\ 0 & c_{1} & -s_{1}\\ 0 & s_{1} & c_{1} \end{array}\right]\times \left[\begin{array}{ccc} c_{2} & 0 & s_{2}\\ 0 & 1 & 0\\ -s_{2} & 0 & c_{2} \end{array}\right],$$

(8) $$l_{z1}={\left[\begin{array}{ccc} 0 & 0 & -l_{1} \end{array}\right]}^{T},$$

Equation 6 becomes

(9) $$A_{E}=\left[\begin{array}{cccc} c_{2} & 0 & s_{2} & -s_{2}l_{1}\\ s_{1}s_{2} & c_{1} & -s_{1}c_{2} & s_{1}c_{2}l_{1}\\ -c_{1}s_{2} & s_{1} & c_{1}c_{2} & -c_{1}c_{2}l_{1}\\ 0 & 0 & 0 & 1 \end{array}\right],$$

The coordination of the elbow *E* (*x*_*E*_, *y*_*E*_, *z*_*E*_) is represented in the 4th column that is,

(10) $$\left[\begin{array}{c} x_{E}\\ y_{E}\\ z_{E} \end{array}\right]=-l_{1}\text{​​}\left[\begin{array}{c} s_{2}\\ -s_{1}c_{2}\\ c_{1}c_{2} \end{array}\right],$$

Therefore,

(11) $$\left[\begin{array}{c} x_{W}\\ y_{W}\\ z_{W} \end{array}\right]=\left[\begin{array}{c} x_{E}\\ y_{E}\\ z_{E} \end{array}\right]+l_{2}\text{​}\left[\begin{array}{c} -s_{2}c_{4}-c_{2}s_{3}s_{4}\\ s_{1}c_{2}c_{4}+(c_{1}c_{3}-s_{1}s_{2}s_{3})s_{4}\\ -c_{1}c_{2}c_{4}+(s_{1}c_{3}-c_{1}c_{2}s_{3})s_{4} \end{array}\right],$$

Finally, we can get the following Equations (12–15).

(12) $$\tan\theta_{1}\;=-\frac{y_{E}}{z_{E}},$$

(13) $$\sin\theta_{2}\;=-\frac{x_{E}}{l_{1}},$$

(14) $$\sin\theta_{3}\;=\frac{\left(x_{E}-x_{W}\right)\text{​}/l_{2}-\sin\theta_{2}\cos\theta_{4}}{\cos\theta_{2}\sin\theta_{4}},$$

(15) $$\cos\theta_{4}\;=\frac{l_{1}^{2}+l_{2}^{2}-R^{2}}{2l_{1}l_{2}},$$

### Estimation of EMG signals from estimated cortical activities

A sparse linear regression method (Toda et al., 2011) was used to estimate filtered EMG signals from the cortical activities estimated over 2240 vertexes.

(16) $$f{\text{EMG}}_{i}\text{​}\left(t+\delta t\right)=\sum_{j\;=\;1}^{N_{\text{source}}}w_{ij}\times J_{j}(t)+\text{bias},$$

Here, *f*EMG_*i*_ describes the *i*-th filtered EMG signal from the cortical activity on the *j*-th vertex (*J*_*j*_). *N*_source_ denotes the number of vertexes used in estimating filtered EMG signals. In this study, since all subjects are right-handed, the cortical activities over 33 vertexes in the left primary motor cortex were used to estimate the filtered EMG signals. The weighting factor *w*_*ij*_ represents the strength influence from the cortical activity on the *j*-th vertex on muscle *i*-th muscle. δt is the delay between the cortical activity of the primary motor cortex and the EMG signals.

### Modular artificial neural network model

**Figure 6:**
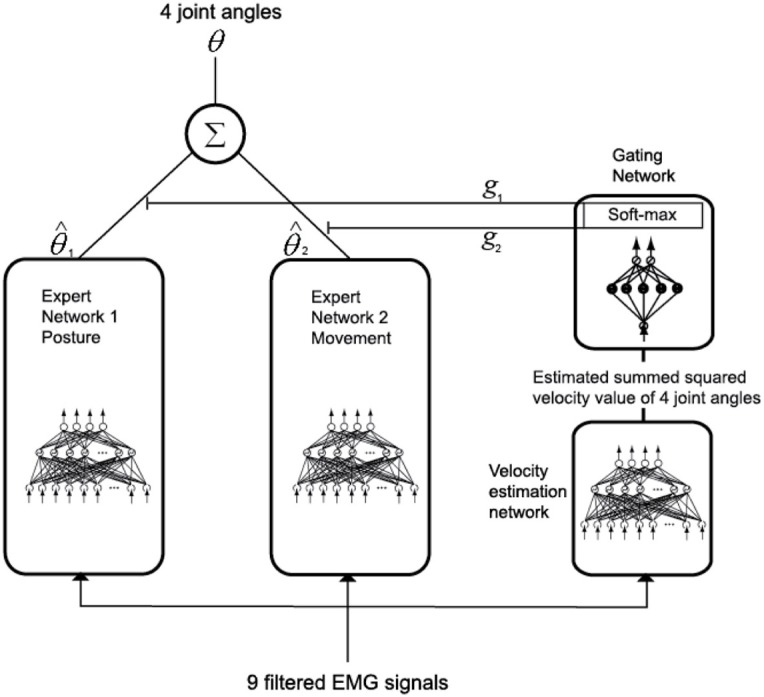
**Joint-angle estimation model with a modular architecture**.

In order to estimate joint angles from the filtered EMG signals, a modular artificial neural network (Jacobs et al., 1991) was used, as shown in Figure 6. Training the data of posture and movement in different networks will improve the accuracy of estimating joint angles compared to training the entire set of data in the same network, since the muscle tension is different in these two cases. Here, posture is defined as the state where the arm of the subject is in contact with a button on the screen, and movement is defined as the condition where the arm of the subject moves from one button to another. If training is done well, a gating network will select one of the two expert networks by its input signal. In this case, one of the two expert networks is used for posture control and the other is used for movement control. Since the gating network determines the output ratio for each expert network depending on its input signal, the sum of the outputs of the gating network should always be equal to 1.

To achieve this, as shown in Equation 17, the output *g*_*j*_ of the gating network, which corresponds to the *j*-th expert network, is normalized by using the soft max activation function.

(17) $$g_{j}=\frac{e^{x_{j}}}{\sum_{i\;=\;1}^{N}e^{x_{i}}},$$

Here, *x*_*i*_ is the value determined by the input signal of the gating network and *N* is the total number of outputs of the gating network. The total output is calculated by multiplying the output of the gating network by the output of each expert network and summing the result, as given in Equation 18.

(18) $$\theta =\sum_{i\;=\;1}^{N}g_{i}{\hat{\theta }}_{i},$$

The gating network and each expert network are trained to maximize the likelihood function lnL (Equation 19) by the back propagation algorithm (Rumelhart et al., 1986).

(19) $$\ln L=\ln\sum_{i\;=\;1}^{N}g_{i}e^{\frac{-{\left\|\theta \;-\;{\hat{\theta }}_{i}\right\|}^{2}}{2\sigma_{i}^{2}}},$$

The update of the weights of the gating network is calculated by a chain rule, as in Equation 20.

(20) $$\frac{\partial \ln L}{\partial x_{i}}=\sum_{i\;=\;1}^{N}\left(g(i|X,{\hat{\theta }}_{i})-g_{i}\right)\text{​},$$

Here, *X* is the input of the gating network, and the posteriori probability $g\left(i|X,{\hat{\theta }}_{i}\right)$ is

(21) $$g\text{​}\left(i|X,{\hat{\theta }}_{i}\right)=\frac{g_{i}e^{\frac{-{\left\|\theta \;-\;{\hat{\theta }}_{i}\right\|}^{2}}{2\sigma_{i}^{2}}}}{\sum_{j\;=\;1}^{N}g_{j}e^{\frac{-{\left\|\theta \;-\;{\hat{\theta }}_{j}\right\|}^{2}}{2\sigma_{j}^{2}}}},$$

The update of the weights of each expert network is calculated by a chain rule as in Equation 22.

(22) $$\frac{\partial \ln L}{\partial {\hat{\theta }}_{i}}=\sum_{i\;=\;1}^{N}g\text{​}\left(i|X,{\hat{\theta }}_{i}\right)\frac{\theta -{\hat{\theta }}_{i}}{\sigma_{i}^{2}},$$

Each network is trained by using the kick-out method (Ochiai and Usui, 1993).

The filtered EMG signals of the nine muscles were used as the input of each expert network model. The summed-squared velocity value of the four joint angles were used as the input of the gating network because when the value of the cortical activities in the primary motor cortex was directly used as the input of the gating network, the gating network could not distinguish between posture and movement. However, when using the summed-squared velocity of the four joint angles as the input, the gating network distinguished posture and movement correctly.

### Analysis

The correlation coefficient (CC) was used to evaluate the similarity between actual and predicted signals. Accuracy was also evaluated using normalized root-mean-square error (nRMSE) between actual and predicted signals, defined as

(23) $$\text{nRMSE}=\sqrt{\frac{\sum_{i\;=\;1}^{n}{\left(y_{i}^{\text{predicted}}-y_{i}^{\text{actual}}\right)}^{2}}{n}}/\left(y_{\max}^{\text{actual}}-y_{\min}^{\text{actual}}\right)\text{​},$$

where for each time i (*i* = 1, 2, …, n), *y*^predicted^_*i*_ is the predicted signal and *y*^actual^_*i*_ is the actual signal, and *y*^actual^_max_ and *y*^actual^_min_ are the maximum and minimum of actual signal, respectively.

## Results

### Estimation result of filtered EMG signals from the cortical activities of the primary motor cortex

The filtered EMG signals were estimated from the cortical activities in the primary motor cortex by using Equation 23. To determine the delay-time parameter, the intracortical microstimulation (ICMS) method (Heusler et al., 2000) was used and the delay time 17 ms was decided when the filtered EMG signals are estimated from the cortical activities of the primary motor cortex.

**Figure 7:**
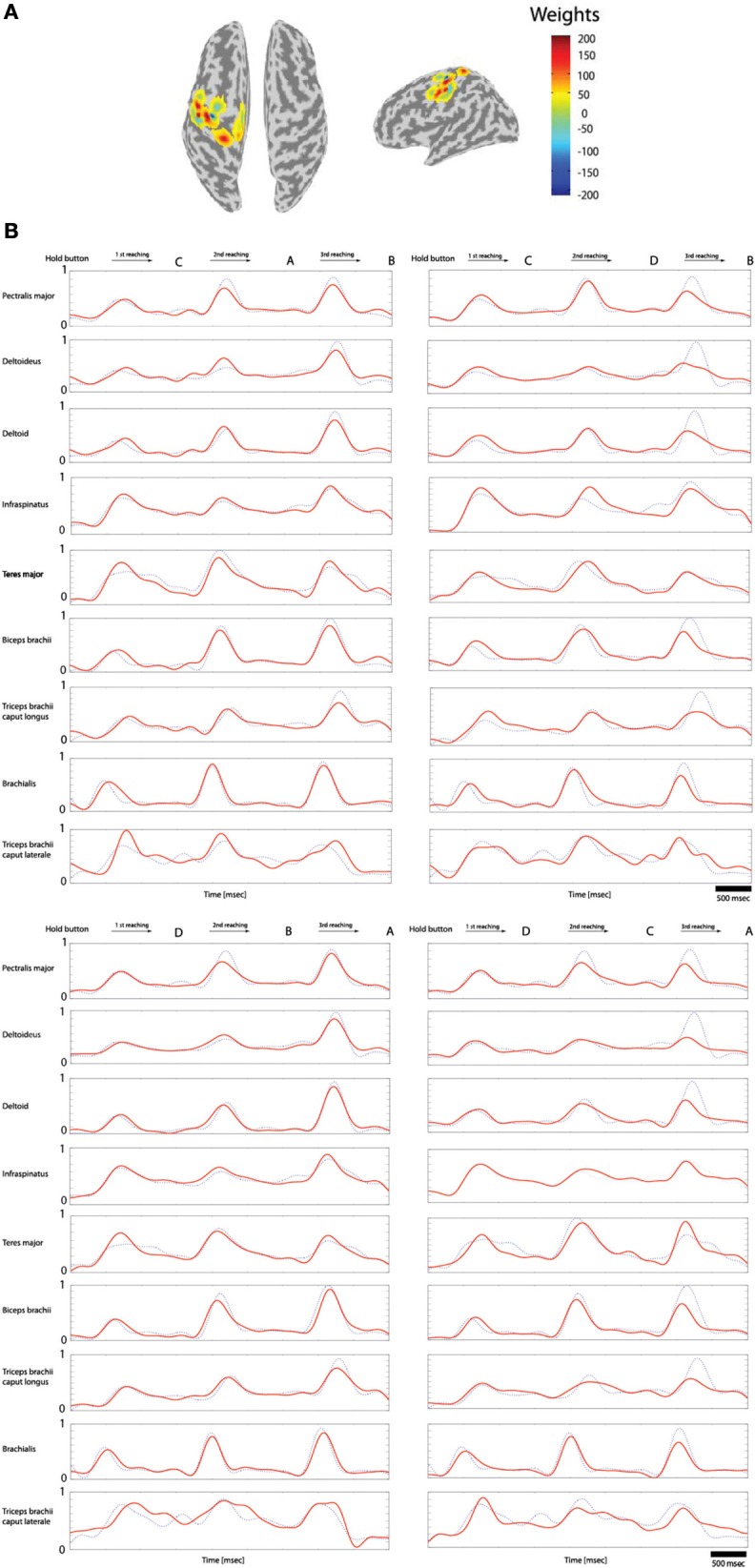
**Reconstruction of the filtered EMG signals from the cortical activities estimated on 33 vertexes in the left primary motor cortex. (A)** The weights of the important features selected by the sparse linear regression to estimated filtered EMG signals from the cortical activities while subject 3 performs four tasks. **(B)** The filtered EMG signals estimated from the selected 20 features. Dotted lines (blue) represents the actual filtered EMG signals, and solid lines (red) show the reconstructed filtered EMG signals (normalized scale).

Of the 70 trials measured for each task (Hold-C-A-B, etc.), 60 trials were used for training data and 10 trials for the test data. The sparse linear regression method has an ability to automatically select only useful features in estimation among all extracted features. Therefore, this method is very strong against the overfitting problem. Figure 7A shows the weights of the selected features in estimating filtered EMG signals from the cortical activities in the left primary motor cortex while subject 3 performs the experimental task. In the case of Figure 7A, 20 vertexes are selected among 33 vertexes located in the left primary motor cortex to estimate filtered EMG signals.

Figure 7B shows the filtered EMG signals of subject 1 estimated from the cortical activities over selected 20 vertexes in the left primary motor cortex. The estimated filtered EMG signals had a CC of 0.827 (±0.10) and nRMSE of 0.142 (±0.38) with the actual EMG signals. Table 2 shows the CC between the actual EMG signals and the reconstructed EMG signals of all of the 5 subjects participated in the experiment. The averaged CC and nRMSE of 5 subjects were 0.851 (±0.11) and 0.233 (±0.17).

**Table 2:** **The correlation coefficient (CC) and normalized root-mean-square error (nRMSE) between the actual EMG signals and the estimated EMG signals**.

**Subject**	**Statistics**	**Task**			
		**CAB**	**CDB**	**DBA**	**DCA**
1	CC	0.82 (0.12)	0.83 (0.12)	0.83 (0.10)	0.83 (0.09)
	nRMSE	0.28 (0.24)	0.14 (0.04)	0.12 (0.07)	0.12 (0.39)
2	CC	0.86 (0.11)	0.88 (0.09)	0.82 (0.07)	0.89 (0.10)
	nRMSE	0.19 (0.25)	0.12 (0.05)	0.18 (0.10)	0.15 (0.05)
3	CC	0.82 (0.11)	0.85 (0.12)	0.86 (0.09)	0.86 (0.09)
	nRMSE	0.14 (0.38)	0.23 (0.24)	0.22 (0.17)	0.23 (0.18)
4	CC	0.88 (0.10)	0.86 (0.13)	0.87 (0.08)	0.84 (0.11)
	nRMSE	0.11 (0.07)	0.12 (0.05)	0.10 (0.06)	0.27 (0.43)
5	CC	0.85 (0.07)	0.86 (0.07)	0.85 (0.12)	0.86 (0.11)
	nRMSE	0.23 (0.43)	0.18 (0.11)	0.22 (0.18)	0.22 (0.42)
Average	CC	0.84 (0.10)	0.85 (0.11)	0.84 (0.10)	0.85 (0.12)
	nRMSE	0.19 (0.06)	0.15 (0.04)	0.16 (0.05)	0.19 (0.06)

### Estimation result of joint angles from filtered EMG signals

**Figure 8:**
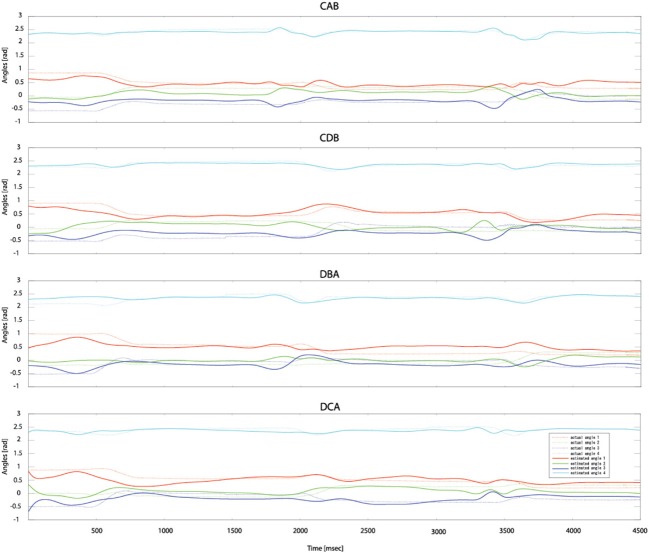
**Estimated joint angles from the cortical activities of the primary motor cortex**. Dashed lines represent the actual joint angles and solid lines show the estimated joint angles.

After measuring 70 trials of the EMG signals and movement trajectories of the subject's arm, 60 trials were used as training data and one trial as test data. The number of training data samples was 1,080,720 (60 trials × 1 kHz × 4.503 s × 4 cases) and the number of test data samples was 180,120 (10 trials × 1 kHz × 4.503 s × 4 cases). In the case of the gating network, the network was trained by the summed-squared velocity value of the four joint angles. However, since this value cannot be used as test data, the velocity values from the filtered EMG signals were estimated. Figure 8 shows the four joint angles of subject 1 estimated from the cortical activities of the primary motor cortex. The CC and nRMSE between the estimated joint angles and the actual joint angles were about 0.817 (±0.10) and 0.212(±0.04). Table 3 depicts the CC between the actual joint angles of 5 subjects and the joint angles reconstructed by the modular artificial neural network model. The averages of the CC and nRMSE of the reconstructed joint angles were 0.807 (±0.10) and 0.176 (±0.29).

**Table 3:** **The correlation coefficient (CC) and normalized root-mean-square error (nRMSE) between the actual joint angles and the joint angles estimated by the modular artificial neural network model**.

**Subject**	**Statistics**	**Task**			
		**CAB**	**CDB**	**DBA**	**DCA**
1	CC	0.81 (0.12)	0.82 (0.11)	0.84 (0.09)	0.80 (0.10)
	nRMSE	0.21 (0.22)	0.19 (0.21)	0.13 (0.12)	0.20 (0.19)
2	CC	0.82 (0.11)	0.75 (0.10)	0.75 (0.09)	0.79 (0.10)
	nRMSE	0.14 (0.05)	0.25 (0.15)	0.22 (0.15)	0.21 (0.11)
3	CC	0.83 (0.11)	0.86 (0.13)	0.79 (0.08)	0.81 (0.08)
	nRMSE	0.24 (0.22)	0.21 (0.24)	0.13 (0.05)	0.22 (0.20)
4	CC	0.78 (0.10)	0.78 (0.14)	0.82 (0.09)	0.88 (0.10)
	nRMSE	0.24 (0.15)	0.23 (0.16)	0.11 (0.07)	0.17 (0.29)
5	CC	0.85 (0.08)	0.82(0.08)	0.84 (0.10)	0.88 (0.10)
	nRMSE	0.25 (0.33)	0.19 (0.21)	0.13 (0.13)	0.20 (0.06)
Average	CC	0.81 (0.08)	0.80 (0.11)	0.80 (0.08)	0.82 (0.08)
	nRMSE	0.21 (0.04)	0.21 (0.02)	0.14 (0.04)	0.20 (0.01)

## Discussion

In this study, the cortical activities on 2240 vertexes were estimated from the EEG signals of 64 channels using the hierarchical Bayesian method. Then, of the estimated cortical activities, only the cortical activities in the left primary motor cortex were used to reconstruct the EMG signals of nine muscles through the sparse linear regression method. When reconstructing EMG signals from the cortical activities, we could determine the delay time between the cortical activities and the EMG signals by searching the correlation of those two signals. However, the pattern of EMG signals has a simple waveform which has one or two peaks, and that of cortical activities is also similar. Thus, in this study, the ICMS method was used to decide the delay time. The delay time of 17 ms found from ICMS method was applied for all the subjects for estimating EMG signals from the cortical activities. In the future, we are going to study whether or not this delay time is effective for individuals in disease state. A modular artificial neural network model was used to estimate four joint angles on the elbow and the shoulder from the estimated EMG signals.

### Why is it important to reconstruct EMG signals from brain signals?

Morrow et al. (Morrow and Miller, 2003) succeeded in reconstructing the EMG signals of the distal forelimb muscles from the 50 M1 neurons of an non-human primate while performing a stereotyped precision grips task. Furthermore, Koike et al. (2006) estimated the EMG signals of seven arm muscles from the neural activities of 18 neurons of an non-human primate during an arm reaching task. Then, three joint angles (two at the shoulder and one at the elbow) were reconstructed from the estimated EMG signals. Similarly, most existing brain-machine interface studies reconstruct EMG signals from the neural activities of the primary motor cortex of non-human primates, by using invasive needle electrodes. In such cases, it is possible to obtain relatively clean brain signals.

When reconstructing EMG signals with non-invasive BMI technologies however, there are several difficulties because the skull, which is an insulator, is located between the brain and the sensors, thus introducing noise. Ganesh et al. (2008) succeeded in reconstructing the EMG signals of two antagonist muscles from fMRI signals measured in the primary motor cortex and pre-motor cortex. EEG signals have good time resolution, but its spatial resolution is poor. Consequently, it is difficult to estimate EMG signals with EEG signals. In this study, spatial resolution is improved by estimating cortical activities over 2240 vertexes from the EEG signals measured over 64 channels through the hierarchical Bayesian method. Among the features being abundant, the sparse linear regression method automatically selects only useful features in reconstructing EMG signals. The proposed method is very robust against the overfitting problem.

When EMG signals are reconstructed from the brain signals, there are several advantages: First, we can reconstruct not only position related information such as hand position but also force related information such as joint torque and stiffness from the estimated EMG signals (Koike and Kawato, 1993, 1994, 1995). For example, when we pick up an object, the brain stabilizes the posture of the arm by controlling muscle tensions. The stiffness is controlled by the co-contraction of the muscles. It is difficult to model this phenomenon by directly estimating the hand position because co-contraction causes different muscle patterns for the same posture. Similarly, when in addition to reconstructing the kinematics of hand motion, we obtain force information such as joint torque and stiffness from the brain signals, it is possible to control a robotic arm based on these information. In such cases it could also be possible to implement a brain-machine interface more compatible with features of the human arm.

Second, by using the estimated EMG signals as the command signals of the FES, we raise the possibility that a paralyzed person could in principle control his arm once we electrically stimulate his paralyzed muscles (Degnan et al., 2002; Uechi et al., 2004). Fagg et al. (2007), without modeling the characteristics of the musculoskeletal system, controlled arm movement by electrical stimulation of arm muscles through FES after reconstructing EMG signals from the neural activities in the primary motor cortex. Furthermore, Moritz et al. (2008), by facilitating the direct control of the stimulation of muscles from the neural activities of the primary motor cortex, made it possible for non-human primates to control bidirectional wrist torques from cortical cells. This research suggests that it may be possible to create more realistic neuro-prostheses. By modeling the musculoskeletal system, we may be able to extend non-invasive brain-machine interfaces to control anthropomorphic robotic devices.

### Which brain part is measured for reconstructing EMG signals?

**Figure 9:**
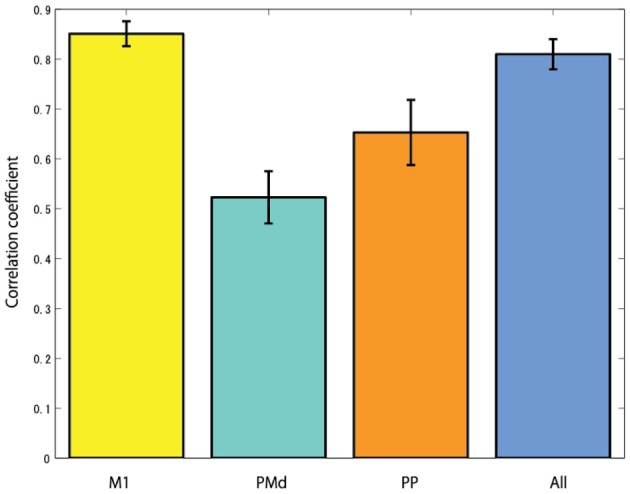
**The correlation coefficients when reconstructing EMG signals from the cortical activities estimated in different brain areas (M1, primary motor cortex; PMd, dorsal premotor cortex; PP, posterior parietal cortex; and All, using all brain areas)**.

The cortical region of choice to harness the control neural signal from seems to be important. In the case of studies using non-human primates, the neural activities of the primary motor cortex are mainly measured to reconstruct EMG signals (Nicolelis et al., 1998; Shoham et al., 2005; Wu et al., 2006). From the research result of Fried et al. (1991), the process of motor related information in the brain is that first the urge to move the arm occurs from the premotor cortex, then the occurred signals goes to the primary motor cortex via the supplementary motor area. The primary motor cortex is the final output part of motor related signals in the brain. The signal is transmitted to the arm muscles through the alpha motor neuron of the spinal cord, and finally it generates arm movement. Anatomically, since the primary motor cortex is linked to the muscles via one or more intermediate neurons, the neural activities of the primary motor cortex have high correlation with muscle activities. Figure 9 shows the CCs when reconstructing EMG signals from the cortical activities in several brain areas. In this study, it is found that when reconstructing EMG signals from the cortical activities estimated in the primary motor cortex, the highest CC is obtained.

## Limitations and future work

There are some limitations with the use of modular neural networks for joint angle estimation. The estimated joint angles have a CC of 0.81 with the actual joint angles. The reason the modular artificial neural network model was used in estimating joint angles is because, in the case of isotonic movement, where force is outputted with a changing length of the muscle, the tension is different depending on the velocity that the muscle flexes or extends. In the case of muscle flexion, the tension decreases as the flex velocity increases. In the case of muscle extension, the tension increases as the extension velocity increases. The performance of estimating joint angles could be improved by training two networks with tension values, which change depending on the velocity, rather than training the data in the same network. One network was used for 0 velocity and the other for movement velocity. When joint angles are estimated from muscle tensions, the muscle tensions for posture have low values. In comparison, the muscle tensions for movement have significantly high values. If we trained these data in the same network, the network would determine that the error of posture data is much lower than that of movement data. Consequently, in the case of posture data, the estimated results are poor. In future work we will use different neural network structures for joint angle estimation. Recent advances in machine learning point at deep learning algorithms and neural networks (Salakhutdinov and Hinton, 2009, 2012) as a possibility for improving feature extraction to reconstruct the joint angles. We plan to explore these new avenues of research.

In this study, five normal subject's joint angles were estimated from EEG signals through EMG signals. In the case of individuals with spinal cord injuries where the pathway between primary motor cortex and muscles was disconnected, there was a necessity of identifying the relationship between EMG signals and joint angles of a normal subject. Then, the EEG signals of an individual with spinal cord injury is connected to EMG signals of the normal subject. In the future, we are going to study more about this topic with individuals with spinal cord injury. Furthermore, there is a possibility of using this proposed method in a study of post-stroke individual where primary motor cortex is not damaged.

### Conflict of interest statement

The author declares that the research was conducted in the absence of any commercial or financial relationships that could be construed as a potential conflict of interest.
