# Erratum: reconstructing four joint angles on the shoulder and elbow from noninvasive electroencephalographic signals through electromyography

**DOI:** 10.3389/fnins.2014.00031

**Published:** 2014-02-21

**Authors:** Kyuwan Choi

**Affiliations:** Psychology Department, Computational Biomedicine Imaging and Modeling, Computer Science, Rutgers UniversityPiscataway, NJ, USA

**Keywords:** EEG, EMG, motor cortex stimulation, joint angles, linear model

In this article, the author's affiliation was wrong. The correct affiliation is as follows,

Department of Computational Brain Imaging, ATR Computational Neuroscience Laboratories, 2-2-2 Hikaridai, Seika-cho, Soraku-gun, Kyoto 619-0288, Japan

e-mail: kyuwanchoi@gmail.com

